# Exploring the potential mechanism of *Polygonatum sibiricum* for Alzheimer’s disease based on network pharmacology and molecular docking: An observational study

**DOI:** 10.1097/MD.0000000000040726

**Published:** 2024-12-27

**Authors:** Liangliang Luo, Yao Pan, Fang Chen, Zhihong Zhang

**Affiliations:** a School of Public Health, Jiangxi Medical College, Nanchang University, Nanchang, China; b Jiangxi Provincial Key Laboratory of Disease Prevention and Public Health, Nanchang University, Nanchang, China.

**Keywords:** active ingredients, Alzheimer’s disease, molecular docking, network pharmacology, polygonatum, potential mechanism

## Abstract

Alzheimer’s disease (AD) is a neurodegenerative disease, and there have been no systematic studies of Polygonatum against Alzheimer’s disease. Therefore, our study will elucidate the mechanism of Polygonatum against AD based on network pharmacology and molecular docking. The active ingredients and corresponding targets of Polygonatum were identified using the traditional Chinese medicine systematic pharmacology database and analysis platform. Disease targets of AD were retrieved from the therapeutic target database, Online Mendelian Inheritance in Man, GeneCards, and Disgenet databases. Using the STRING database, we constructed protein interaction networks and performed gene ontology functional enrichment analysis as well as Kyoto encyclopedia of genes and genomes pathway enrichment analysis on common targets. We then drew drug-component-target-pathway-disease network maps using Cytoscape 3.10.1 software and validated the molecular docking using AutoDock4. A total of 10 active ingredients and 108 common targets were screened from Polygonatum, 29 genes (including AKT1 and STAT3) were identified as core genes. According to gene ontology analysis, the core targets were found to be mainly involved in signal transduction, positive regulation of gene expression, negative regulation of the apoptotic process, and so on. The Kyoto encyclopedia of genes and genomes analysis revealed that the signaling pathways comprised pathways in cancer, pathways of neurodegeneration – multiple diseases, and PI3K-Akt signaling pathway. The molecular docking results indicated that 10 of active ingredients from Polygonatum exhibited strong binding affinity with the 6 core targets that were screened before. The activity of Polygonatum against AD could be attributed to the regulation of multiple biological effects via multi-pathways (pathways in cancer, pathways of neurodegeneration – multiple diseases, and PI3K-Akt signaling pathway). The binding activities were estimated as good level by molecular docking. These discoveries disclosed the multi-component, multi-target, and multi-pathway characteristics of Polygonatum against AD, providing a new strategy for such medical problem.

## 1. Introduction

Alzheimer’s disease (AD) is a neurodegenerative condition with gradual onset and slow progression. It is characterized by progressive cognitive impairment and mobility difficulties.^[1]^ The impact of this disease on the daily lives of middle-aged and elderly people is significant, and its incidence increases as the population ages.^[2]^ The incidence of Alzheimer’s disease almost doubles every 5 years in people over the age of 65.^[3]^ The diagnosis of Alzheimer’s disease is based on cognitive and behavioral scores, brain imaging, and the analysis of various biomarkers.^[4]^ Additionally, AD is characterized by a prolonged asymptomatic preclinical phase.^[5]^ It is important to maintain objectivity in the evaluation of these features. Currently, drug therapy remains the primary method of treating symptoms. Although the number of people with Alzheimer’s disease is increasing worldwide, only 5 treatments have been approved: memantine, rivastigmine, galantamine, donepezil, and combination therapy.^[6]^ The main agents in this combination are memantine and donepezil. Some immunotherapies have had to temporarily halt related drug development due to their marked acceleration of cognitive deterioration, despite significantly reducing cerebrospinal fluid amyloid beta concentrations.^[7]^ As a result, there is an urgent need for new therapeutic options.

Polygonatum is a medicinal plant that belongs to the Polygonatum genus of the Liliaceae family. The dried rhizome is the medicinal part of the plant and is also used as a food source.^[8]^ Anhui is one of the main production areas for Polygonatum in China.^[9]^ Polygonatum was first published in the “Famous Doctors” Record’. Its functions include tonifying the spleen and kidney, replenishing qi and nourishing yin, dispelling wind, and removing dampness.^[10]^ Polygonatum contains polysaccharides, saponins, flavonoids, amino acids, quinone compounds, vitamins, alkaloids, and a variety of trace elements. It has been shown to regulate immunity, improve memory, act as an antioxidant, delay aging, protect the cardiovascular system, lower blood sugar levels, regulate lipid levels, and exhibit anti-properties.^[11–13]^ Previous studies have demonstrated that Polygonatum can enhance cognitive ability in models of Alzheimer’s disease^[14]^ and mitigate β-amyloid-induced neurotoxicity.^[15]^ However, its therapeutic mechanism has not been extensively studied, and further research is required.

Chinese medicine has thousands of years of clinical experience in treating complex diseases. However, due to its multi-component, multi-target, and synergistic nature, the material basis and mechanism of action of Chinese medicine are not yet clear. This complexity has hindered the modernization of traditional Chinese medicine and has not convinced many domestic and foreign doctors. In 1999, Professor Li Shao proposed the hypothesis of an association between traditional Chinese medicine (TCM) and biomolecular networks. This led to the development of the concept and method of “network target,”^[16]^ followed by the core theory of network pharmacology – the “network target” theory in the TCM field.^[17]^ In 2007, British pharmacologist Hopkins proposed the concept of “network pharmacology.”^[18]^ He defined it as a branch of pharmacology that uses networks to analyze the synergistic relationship between drugs, diseases, and targets. This approach is “multi-component, multi-target, multi-pathway” and is the inevitable product of drug systematic research in the era of artificial intelligence and big data. Cyberpharmacology acknowledges that the development and progression of the disease is a complex and dynamic process that results from a dysfunction in the organism’s intricate network, which may involve multiple biological processes (BPs). Thus, it appears that the previous notion of “single drug, single target” is not justifiable. Instead, the focus should be on examining the molecular correlation between drugs and patients from a systemic level and the entire biological network. Currently, network pharmacology is extensively employed to identify active compounds in traditional Chinese medicine, interpret the overall mechanism of action, and study drug combinations and formulae. This approach offers a new perspective for investigating the vast and intricate system of traditional Chinese medicine and significantly contributes to its modernization.

Molecular docking is a computer-based method used for structural studies.^[19]^ It involves simulating the geometry of molecules and calculating intermolecular forces through stoichiometry to study intermolecular interactions. The method searches for low-energy binding modes between a small molecule (or ligand) and the active site of a macromolecule (or receptor) of known structure. The popularity of molecular docking has been facilitated by the accessibility of small molecule ligands, large molecule protein structures, and the growth of computer power. This research field is full of opportunities and challenges.^[20]^ This study aims to investigate the molecular mechanism of Polygonatum to Alzheimer’s disease using a network pharmacological approach. The results will be validated through simulated molecular docking techniques, providing a basis for future studies and clinical applications.

## 2. Materials and methods

**Figure 1.**
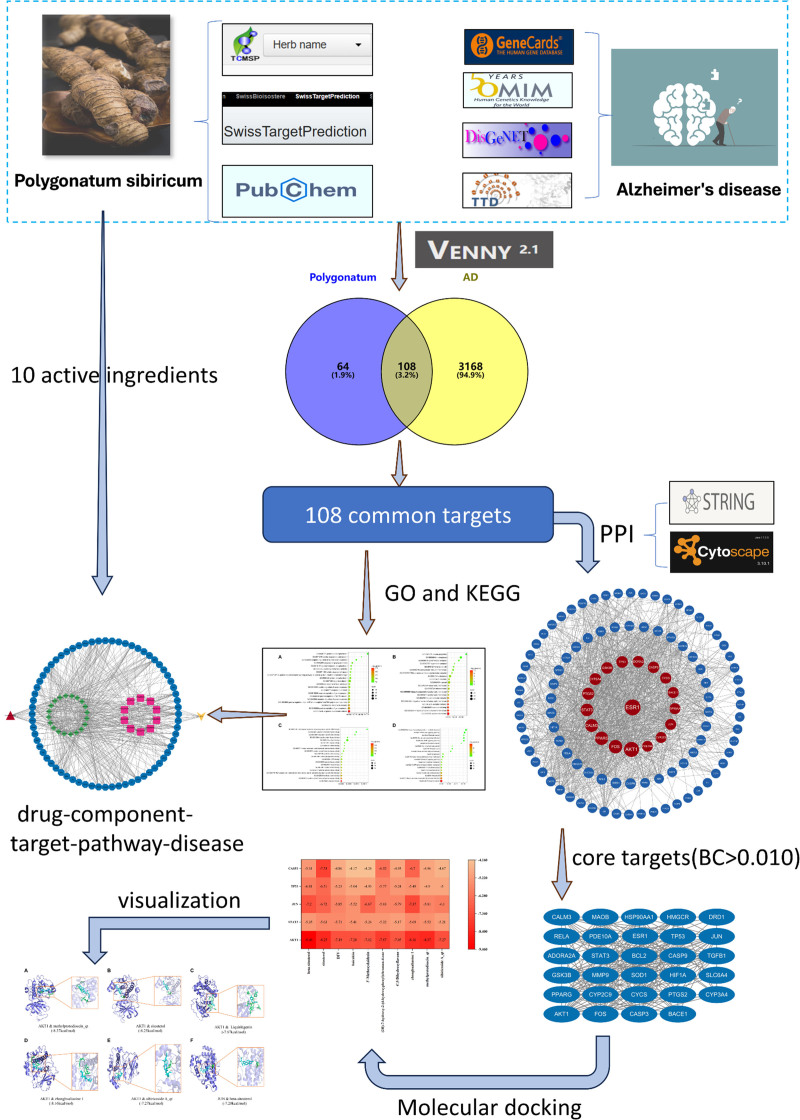
The whole research process. BC = betweenness centrality, GO = gene ontology, KEGG = Kyoto encyclopedia of genes and genomes, PPI = protein–protein interaction.

Figure 1 shows the entire research process.

### 2.1. Screening of active components and potential targets in Polygonatum

The traditional Chinese medicine systematic pharmacology database and analysis platform (TCMSP) is an online database for chemical studies of TCMs.^[21]^ It provides extensive information on the chemistry and pharmacology of TCMs. The screening criteria used were oral bioavailability of at least 30% and drug-likeness of at least 0.18.^[22]^ Oral bioavailability is the measure of a drug’s ability to reach an effective concentration in the bloodstream after entering the body, while drug similarity indicates the likelihood of a compound becoming a drug. The targets obtained were imported into the UniProt database to obtain the corresponding gene names.^[23]^ For targets not found in the TCMSP database, the component’s mol2 file can be downloaded from the TCMSP database and converted to the corresponding SMILES number (Simplified Molecular Input Line Entry System, is a notation that converts chemical structures into ASCII strings, allowing for the processing and manipulation of molecular structures in computer programs.) using an online SMILES conversion website. The SMILES number can then be used to predict possible targets in the Swiss Target Prediction database.^[24]^ A filtering condition of “probability” > 0 was used. If the number for the component cannot be queried, download the 2D structure of the component in SDF format from the PubChem database.^[25]^ Predict the structure in the Swiss Target Prediction database and integrate all obtained targets.

### 2.2. Screening for targets related to Alzheimer’s disease

The GeneCards,^[26]^ DisGeNet,^[27]^ Online Mendelian Inheritance in Man,^[28]^ and therapeutic target database^[29]^ databases were searched using the keyword “Alzheimer’s disease” to identify disease targets.

### 2.3. Accessing common targets for Polygonatum and Alzheimer’s disease

Access to common drug and disease targets with Venny 2.1.0.

### 2.4. Building a PPI protein interaction network map and screening core targets

Import common targets into the STRING database,^[30]^ select “Multiple Proteins” and set the species to “Homo sapiens” before starting the search, and set the confidence level to “high confidence (0.700)” in the “Settings” section after generating the original network graph. After generating the original network graph, set the confidence level to “high confidence (0.700)” in the “Settings” section, and check the “hide disconnected nodes in the network” box. Hide disconnected nodes in the network, generate the protein–protein interaction (PPI) network diagram, and download the TSV file. To observe the protein interactions more intuitively, the results were imported into Cytoscape 3.10.1 to visualize the PPI network plots. Cytoscape’s CytoNCA plug-in was used to screen the core targets, and the core targets were obtained by setting BetweennessCentrality > 0.010.

### 2.5. Gene ontology and Kyoto encyclopedia of genes and genomes pathway analysis

The common targets were imported into the David database^[31]^ for gene ontology (GO) enrichment analysis and Kyoto encyclopedia of genes and genomes (KEGG) pathway analysis. The top 20 results of the analysis were selected. The 20 items’ results were imported into the online bioinformatics analysis and visualization cloud platform Microbiosense for visualization.

### 2.6. Constructing drug-component-target-pathway-disease maps

The data on effective active ingredients, common targets, and the first 20 results from KEGG pathway analysis were collected and imported into Cytoscape software to create maps of Polygonatum-ingredient-target-pathway-Alzheimer’s disease.

### 2.7. Docking of molecules

Molecular docking is a technique used to confirm the association between ingredients and targets. To begin, the mol2 format file of the active ingredient was obtained from the TCMSP database, while the pdb format file of the core target protein was obtained from the RCSB Protein Data Bank.^[32]^ The selection of proteins should adhere to the following criteria as closely as possible: human origin, complete sequence conformation, small molecule ligand information in the structural complex, resolution of the conformation ≤ 3 Å, and determination of protein structure through X-ray crystallographic methods. The mol and pdb files were imported into AutoDocktools software.^[33]^ The files were then exported as pdbqt format files for both the target proteins and small molecule ligands. Before export, the files underwent dehydration, hydrogenation, and charge calculation for the target proteins, and hydrogenation and detection of torsion bonds for the small molecule ligands. After reviewing the literature and screening the core target proteins, we performed semiflexible docking with AKT1, STAT3, JUN, TP53, and CASP3, respectively, using the active ingredients in turn. The molecular docking results were expressed by heat maps, which showed the difference in binding energies. A binding energy < 0 indicates that the ligand and protein can be docked in the natural state, while a binding energy < ‐1 indicates a strong affinity between the ligand and protein. The binding energy of 2 kcal/mol indicates that the ligand and protein can be docked in their natural state. A binding energy of ‐1.2 kcal/mol suggests a good docking result, while a binding energy of ‐7.0 kJ/mol indicates strong binding activity (1 kcal = 4.186 kJ).^[34,35]^ The lower the binding energy, the better the binding activity and the more stable the structure of the binding complex. The docking result file is converted to a pdbqt format file using OpenBabelGUI and then imported into PyMol2.5^[36]^ for visualization. The first 6 effective dockings that combine well are selected to show the results of this docking visualization.

## 3. Results

### 3.1. Screening of active ingredients and potential targets in Polygonatum

The TCMSP database yielded 12 active ingredients meeting the screening conditions of oral bioavailability ≥ 30% and drug-likeness ≥ 0.18. However, 2 of these ingredients were isolated nodes in the subsequent construction of PPI network diagrams. As a result, only 10 effective active ingredients were retained and they were shown in Table 1. By summarizing the TCMSP database and the Swiss Target Prediction database, 253 targets were obtained, and 172 drug targets were obtained by de-weighting. After summarizing the TCMSP and Swiss Target Prediction databases, we obtained 253 targets, of which 172 were de-emphasized as drug targets.

**Table 1:** Ten active ingredients to treat Alzheimer’s disease.

NO	ID	Active ingredient
1	MOL000358	Beta-sitosterol
2	MOL000359	Sitosterol
3	MOL001792	DFV
4	MOL002714	Baicalein
5	MOL002959	3′-Methoxydaidzein
6	MOL004941	Liquiritigenin
7	MOL006331	4′,5-Dihydroxyflavone
8	MOL009766	Zhonghualiaoine 1
9	MOL003889	Methylprotodioscin_qt
10	MOL009760	Sibiricoside A_qt

### 3.2. Access to disease-related targets and common targets

**Figure 2.**
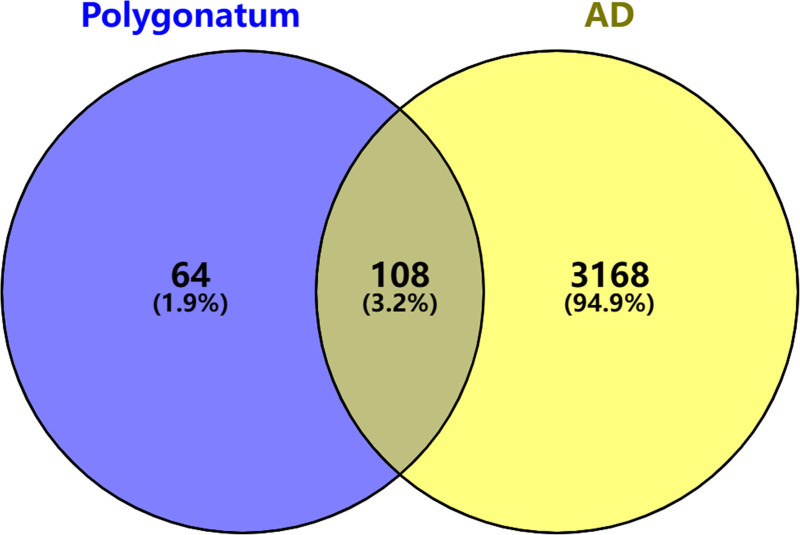
One hundred eight common targets of polygonatum and AD. AD = Alzheimer’s disease.

A total of 4217 disease targets were identified, with 1725 targets in GeneCards, 1848 targets in DisGeNet, 546 targets in Online Mendelian Inheritance in Man, and 98 targets in therapeutic target database. After weight removal, there were 3276 targets. The drug targets and disease targets were intersected using Venny 2.1.0, resulting in 108 common targets, and the results are shown in Figure 2.

### 3.3. Construction of PPI protein interaction network map and screening of core targets

**Figure 3.**
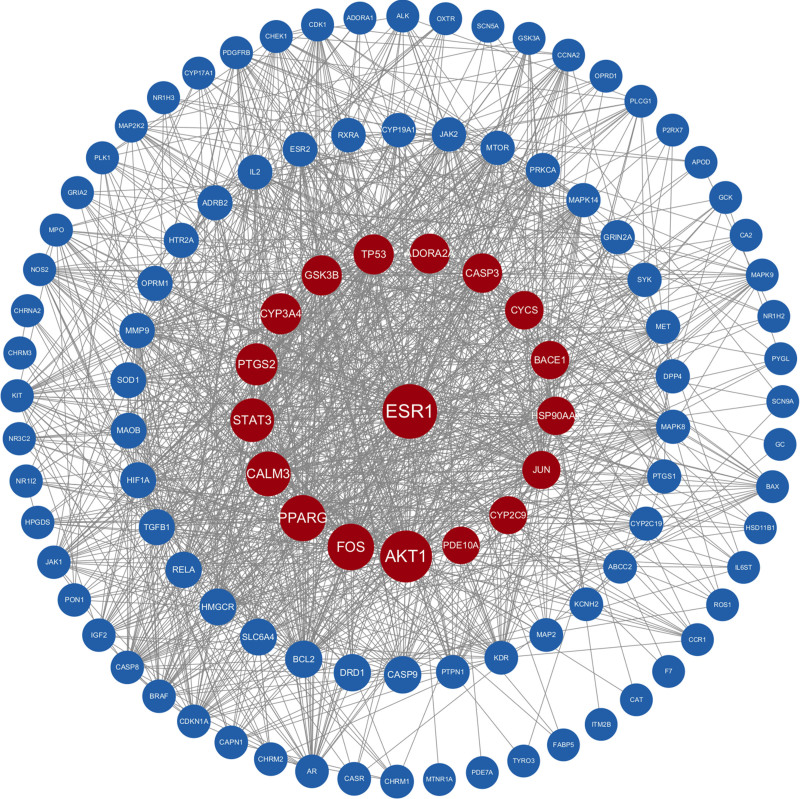
PPI protein interactions of 108 common targets. PPI = protein–protein interaction.

**Figure 4.**
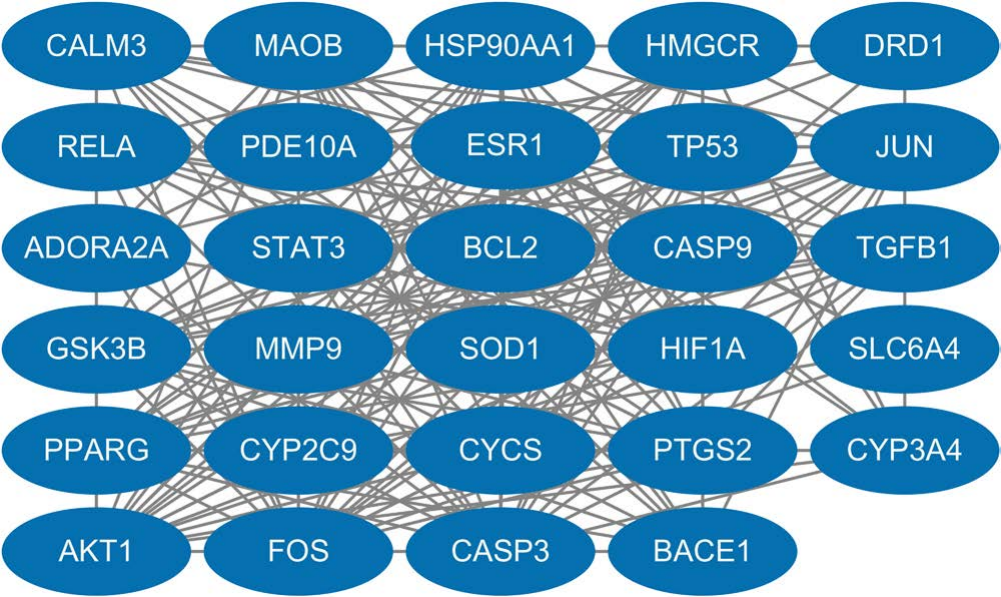
Set BC value > 0.010 to get 29 core targets. BC = betweenness centrality.

The STRING web platform’s network graph comprises 108 nodes and 1194 edges, with an average node degree of 22.1 and an average local clustering coefficient of 0.6. The expected number of edges for this graph was calculated to be 486. The results were exported in TSV format and imported into Cytoscape to plot the PPI graph of the 108 common targets, as shown in Figure 3. Twenty-nine core targets, including TP53 and AKT1, were screened using Cytoscape’s plug-in CytoNCA with a BetweennessCentrality threshold of 0.010. These targets were identified as crucial for the treatment of Alzheimer’s disease (refer to Table 2 and Fig. 4).

**Table 2:** Twenty-nine core targets for treating Alzheimer’s disease.

NO	Gene name	Entry	Name
1	ESR1	P03372	Estrogen receptor 1
2	AKT1	P31749	AKT serine/threonine kinase 1
3	FOS	P01100	Fos proto-oncogene, AP-1 transcription factor subunit
4	PPARG	P37231	Peroxisome proliferator activated receptor gamma
5	CALM3	P0DP25	Calmodulin 3
6	STAT3	P40763	Signal transducer and activator of transcription 3
7	PTGS2	P35354	Prostaglandin-endoperoxide synthase 2
8	CYP3A4	P08684	Cytochrome P450 family 3 subfamily A member 4
9	GSK3B	P49841	Glycogen synthase kinase 3 BETA
10	TP53	P04637	Tumor protein P53
11	ADORA2A	P29274	Adenosine A2a receptor
12	CASP3	P42574	Caspase 3
13	CYCS	P99999	Cytochrome c, somatic
14	BACE1	P56817	Beta-secretase 1
15	HSP90AA1	P07900	Heat shock protein 90 alpha family class A member 1
16	JUN	P05412	Jun proto-oncogene, AP-1 transcription factor subunit
17	CYP2C9	P11712	Cytochrome P450 family 2 subfamily C member 9
18	PDE10A	Q9Y233	Phosphodiesterase 10A
19	CASP9	P55211	Caspase 9
20	DRD1	P21728	Dopamine receptor D1
21	BCL2	P10415	BCL2 apoptosis regulator
22	SLC6A4	P31645	Solute carrier family 6 member 4
23	HMGCR	P04035	3-Hydroxy-3-methylglutaryl-CoA reductase
24	RELA	Q04206	RELA proto-oncogene, NF-kB subunit
25	TGFB1	P01137	Transforming growth factor beta 1
26	HIF1A	Q16665	Hypoxia inducible factor 1 subunit alpha
27	MAOB	P27338	Monoamine oxidase B
28	SOD1	P00441	Superoxide dismutase 1
29	MMP9	P14780	Matrix metallopeptidase 9

### 3.4. GO annotation and KEGG enrichment analysis of common targets

**Figure 5.**
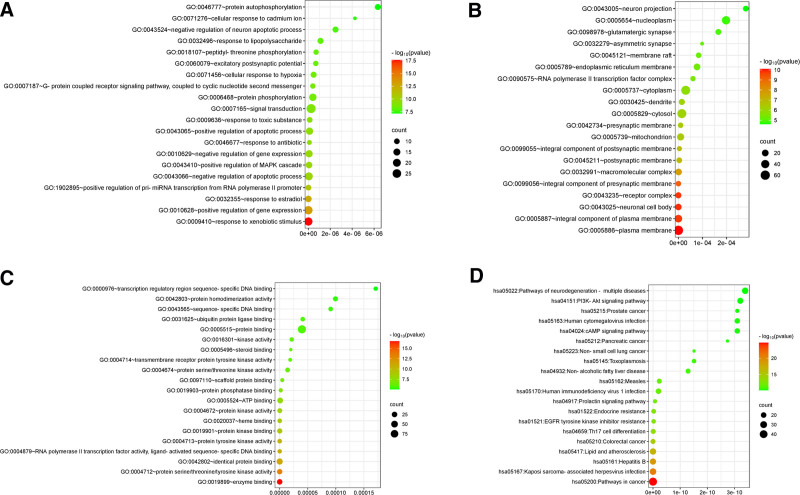
GO and KEGG enrichment analysis of common targets. (A) BP for the first 20 *P* values; (B) CC for the first 20 *P* values; (C) MF for the first 20 *P* values; (D) KEGG for the first 20 *P* values. BP = biological process, CC = cellular component, GO = gene ontology, KEGG = Kyoto encyclopedia of genes and genomes, MF = molecular function.

**Figure 6.**
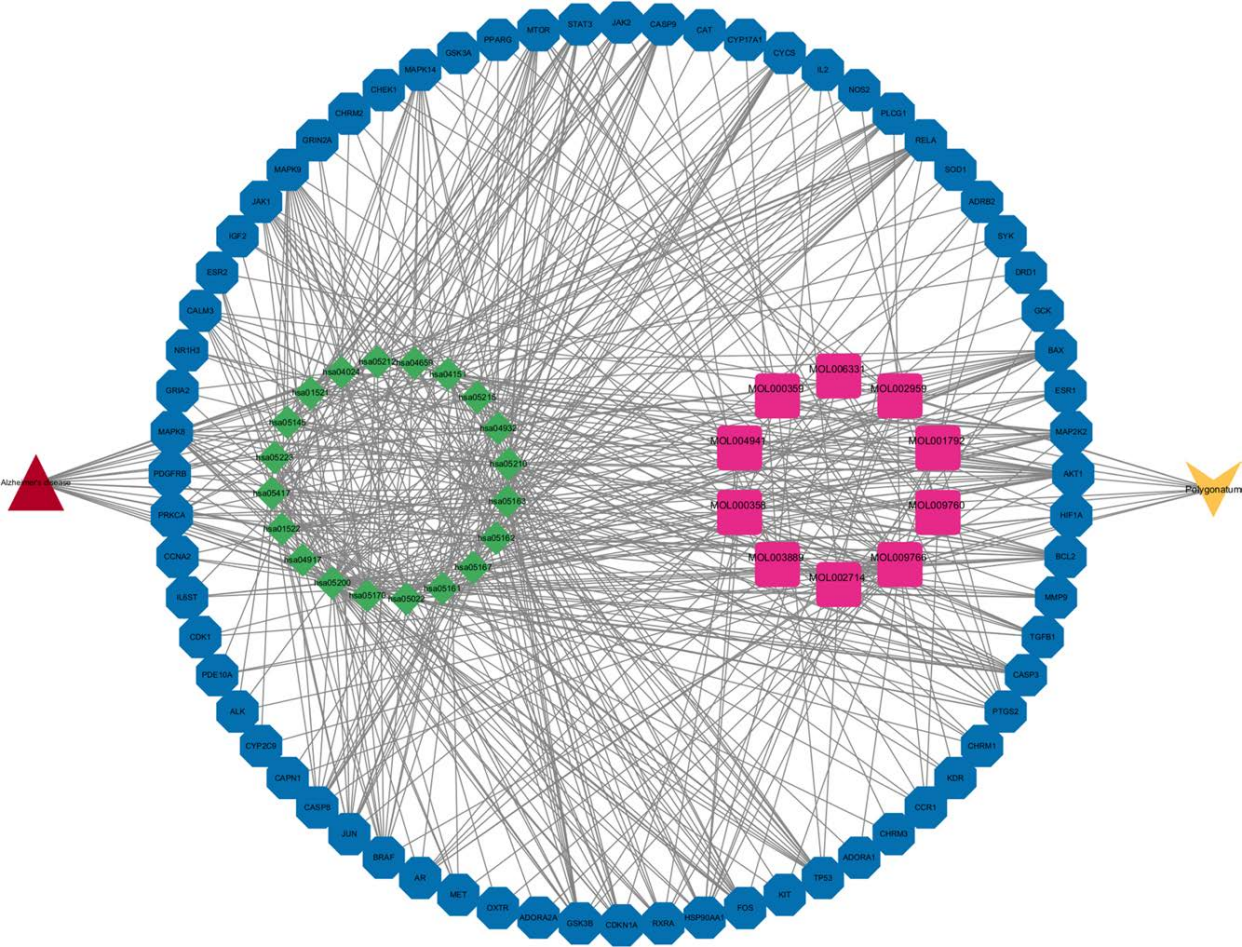
Polygonatum-component-target-pathway-Alzheimer’s disease map.

Based on the DAVID data platform, GO and KEGG enrichment analyses were performed on 108 common targets. Based on *P* < .05, a total of 592 items were screened by GO function enrichment analysis, including 433 items of BP, 64 items of cellular component, 95 items of molecular function, and 155 pathways were screened by KEGG enrichment analysis. The first 20 results of the 4 analyses are shown in Figure 5. The BPs mainly involve signal transduction, positive regulation of gene expression, positive regulation of transcription from RNA polymerase II promoter, response to xenobiotic stimulus, and negative regulation of the apoptotic process. The cellular components include the plasma membrane, cytosol, cytoplasm, and nucleus. About AD, Polygonatum is mainly associated with the function of molecules such as protein binding, identical protein binding, ATP binding, and enzyme binding. The KEGG enrichment analysis screened 155 pathways, including pathways in cancer, pathways of neurodegeneration – multiple diseases, lipid and atherosclerosis, and the PI3K-Akt signaling pathway. The active ingredients of Polygonatum are suggested to have the potential to treat Alzheimer’s disease through important signaling pathways. Additionally, a diagram was created to more intuitively link the drug components to the disease, as shown in Figure 6.

### 3.5. Validation of molecular docking

**Figure 7.**
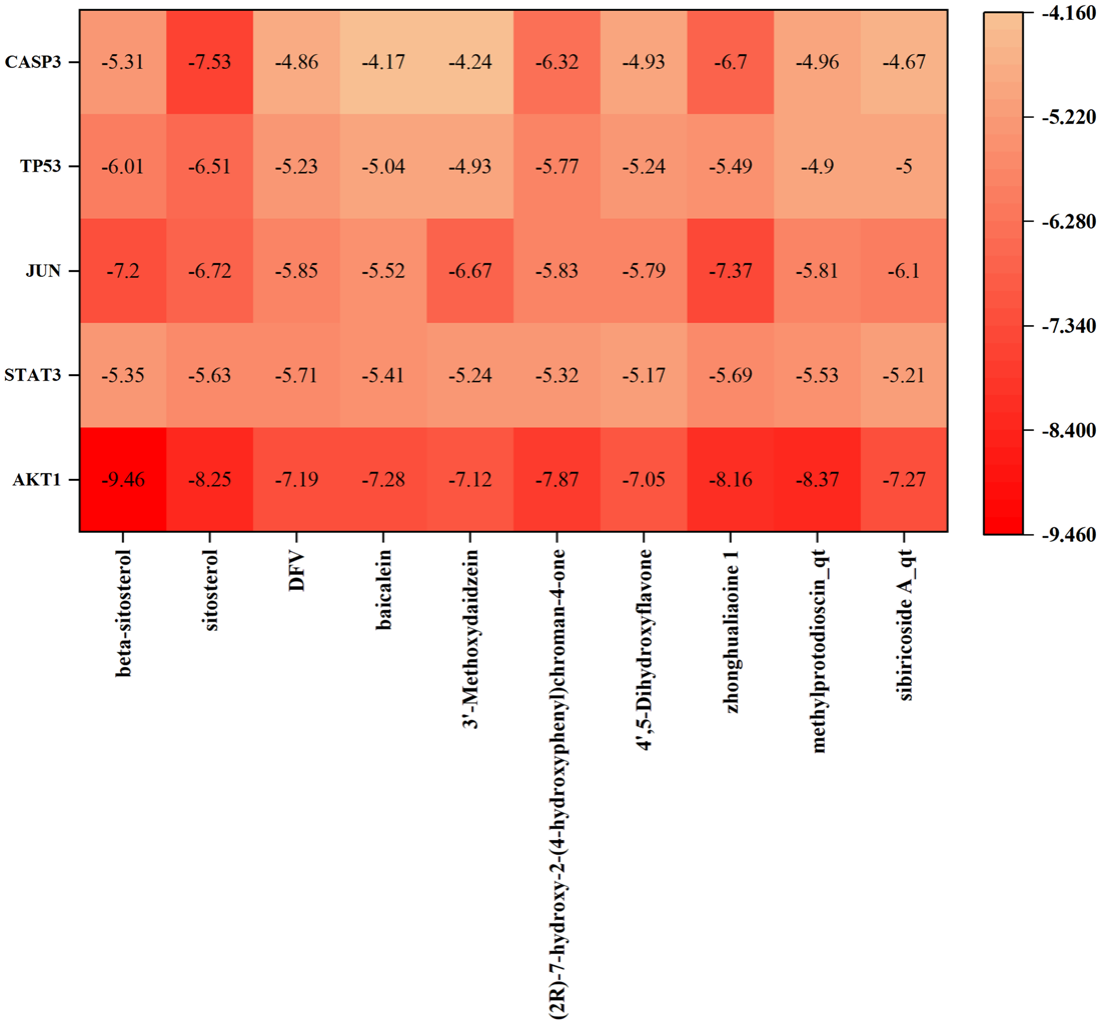
Heat map of molecular docking binding energy.

**Figure 8.**
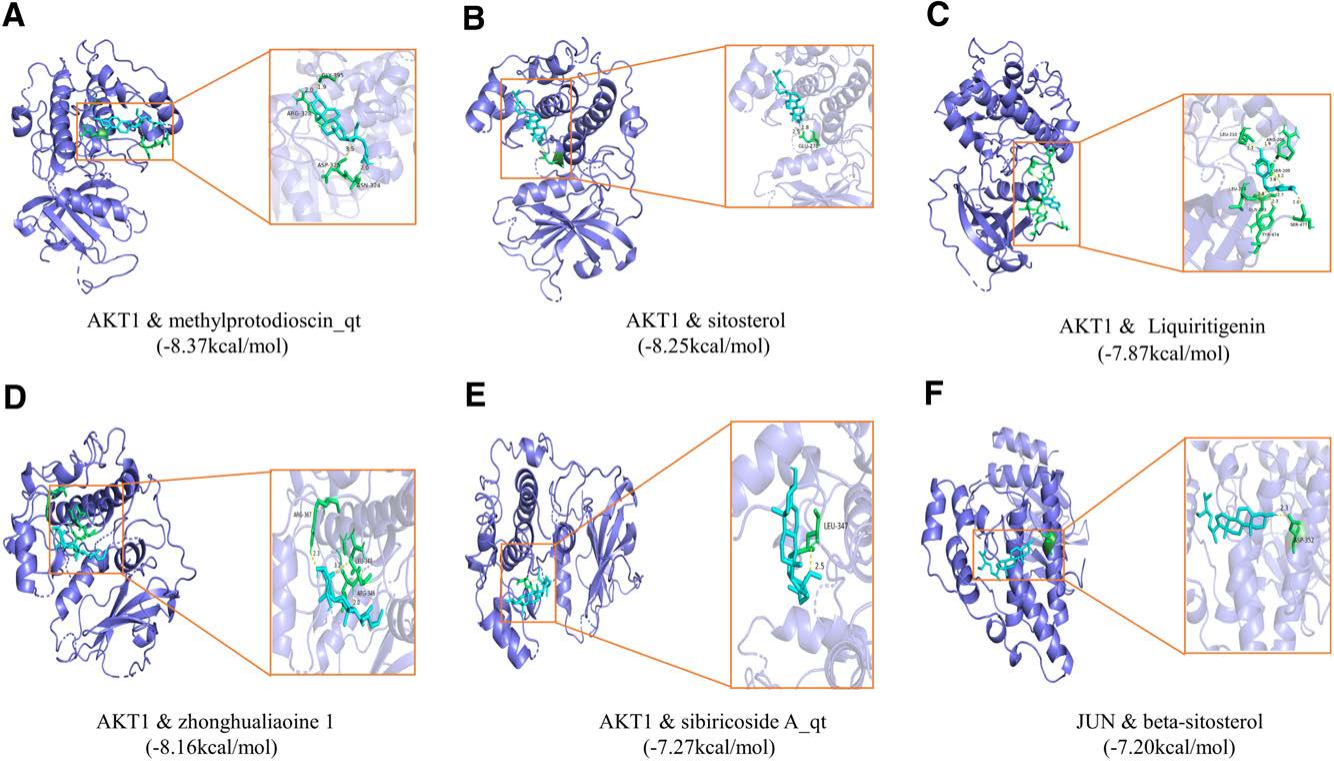
Schematic representation of the molecular docking visualization.

Molecular docking is a computational technique that predicts the interactions between small-molecule ligands and large-molecule protein receptors. It uses software to simulate the binding of these molecules and infer affinity profiles from binding energies. Six core target proteins, namely AKT1, STAT3, JUN, TP53, and CASP3, were selected as representative examples based on the description in Section 2.7. The active ingredients of Polygonatum, including beta-sitosterol, sitosterol, DFV, baicalein, 3′-methoxydaidzein, liquiritigenin, 4′,5-dihydroxyflavone, zhonghualiaoine 1, methylprotodioscin_qt, sibiricoside A_qt, were subjected to molecular docking for a total of 50 times. The binding energy is shown in Figure 7. Figure 8 shows the 6 docking sites with the lowest binding energy. The thermogram also demonstrates the feasibility of Polygonatum for treating Alzheimer’s disease.

## 4. Discussions

Alzheimer’s disease is a progressive neurodegenerative condition that involves multiple BPs. Single-targeted therapy is no longer in fashion. TCM treatment takes a holistic approach, using a multi-targeted and multi-pathway therapeutic approach. This provides a new way of thinking about the treatment of Alzheimer’s disease. This study screened 10 active ingredients from Polygonatum using Network pharmacology. The active ingredients include methylprotodioscin_qt and sibiricoside A_qt of saponins, beta-sitosterol, sitosterol, and zhonghualiaoine1 of phytosterols, and flavonoids. Additionally, liquiritigenin, 4′,5-dihydroxyflavone, DFV, baicalein, and 3′-methoxydaidzein were identified. Saponins are natural plant compounds that can be classified into 2 main groups: triterpene saponins and steroidal saponins.^[37]^ These compounds have various pharmacological properties, such as promoting learning and memory,^[38]^ reducing inflammation and oxidative stress,^[39,40]^ lowering Aβ levels, inhibiting tau protein hyperphosphorylation,^[41,42]^ and decreasing apoptosis in neuronal cells.^[43,44]^ Screening based on Ellman method and HPLC-QTOF MS technique revealed that Zhimai steroidal saponins exhibit moderate or weak AChE inhibitory activity,^[45]^ indicating their potential as an anti-AD drug. On the other hand, Diosgenin exerts its therapeutic effect on AD by modulating NOX 4/NOX 4-mediated oxidative stress and inflammatory responses.^[46]^ Phytosterols are a class of natural compounds that cannot be synthesized by the human body. They play an important role in regulating cholesterol levels, combating atherosclerosis, and maintaining brain health.^[47,48]^ In this study, β-sitosterol, a significant dietary phytosterol, inhibited cholinesterase activity in the hippocampus and frontal cortex and decreased the free radical load in brain tissue.^[49]^ The mechanism of neuroinflammatory action during the course of AD has not been fully elucidated.^[50]^ However, the inflammatory process in AD certainly involves several proinflammatory factors, such as cytokines (e.g., IL-6, TNF-α), transcription factors (e.g., NF-κB), and enzymes (e.g., COX-2).^[51]^ Experimental studies have shown that β-sitosterol can induce anti-neuro injury effects by inhibiting COX-2, IL-6, and NO.^[52]^ Tau proteins are phosphorylated proteins found in the normal human brain. In AD patients’ brains, the number of phosphorylated Tau proteins per molecule can increase to 5–9, compared to the normal 2 to 3, causing them to lose their normal biological functions. In a cellular assay conducted in vitro, the resistance of cell membranes to oxidative stress and lipid peroxidation mediated by glucose oxidase was enhanced by the addition of beta-sitosterol.^[53]^ Several studies have demonstrated a close relationship between mitochondrial dysfunction and the development of AD.^[54–56]^ Beta-sitosterol increases ATP levels in the inner mitochondrial membrane, which is beneficial for AD. Cholesterol has been found to play a role in amyloid-β-producing enzyme activity.^[53]^ Low levels of cholesterol inhibit Aβ accumulation, and β-sitosterol significantly reduces serum cholesterol levels.^[57,58]^ Additionally, an experimental study found that chronic intake of phytosterols in mice caused irreversible accumulation of phytosterols in the brain.^[59]^ Flavonoids are the third group of anti-AD potentials in Polygonatum. Baicalein, a flavonoid, has been shown to have neuroprotective effects both in vivo and ex vivo.^[60]^ It inhibits disease-associated amyloid production and deposition, reduces oxidative stress and inflammatory response, promotes neural differentiation, and increases resistance to apoptosis.^[61]^ Xie et al discovered that baicalein stimulates the phenotypic transformation of activated microglia through the CX3CR1/NF-κB pathway and reduces neuroinflammatory responses, improving learning ability in model mice.^[62]^ Ji et al demonstrated that baicalein inhibits Aβ25–35-induced oxidative damage, thereby reducing apoptosis.^[63]^ Research has demonstrated that baicalein can reverse memory and cognitive deficits induced by Aβ by repairing damaged neurons.^[64]^ Impaired cognitive function in the brain is closely related to abnormal functioning of the cholinergic system. Clinical practice has utilized a variety of acetylcholinesterase inhibitor drugs, and preclinical studies have provided ample evidence that restoration of the cholinergic system not only improves cognitive function symmetrically but also attenuates the pathological features of AD, such as β-amyloid aggregation and hyperphosphorylation of tau proteins.^[65]^ In contrast, liquiritigenin prevents the formation of Tau amyloidogenic fibrils and the exposure of hydrophobic plaques.^[66]^ Additionally, liquiritigenin significantly reduces oligomeric levels of Aβ proteins in the mouse brain in related mouse experiments, although it does not alter β-amyloid precursor protein levels.^[67]^ Liquiritigenin improves scopolamine-induced learning and memory deficits by enhancing and protecting the BDNF/ERK/CREB signaling pathway.^[68]^ Yang et al discovered that 4′,5-dihydroxyflavone significantly increased the survival of PC12 cells after Aβ25–35 attack and elevated the Ca2+ concentration in these cells. This suggests that 4′,5-dihydroxyflavone may have neuroprotective effects through dopaminergic synaptic pathways.^[69]^ Among the available treatments for Alzheimer’s disease are acetylcholinesterase inhibitors, such as donepezil, galantamine, and others, which have been approved for use in treating mild, moderate, and severe cases of Alzheimer’s disease. A significant contributor to the pathogenesis of Alzheimer’s disease is the deficiency of the neurotransmitter acetylcholine. Acetylcholinesterase inhibitors play a symptomatic therapeutic role in a variety of ways, including the inhibition of AChE and the protection of cells from β-amyloid-induced damage and free radical toxicity.^[70]^ For example, in a study exploring and validating the mechanism of donepezil in Alzheimer’s disease, network pharmacology and molecular docking techniques were also employed. The results demonstrated that donepezil is strongly associated with the regulation of chemosynaptic transmission pathways and trans-synaptic signaling pathways. Additionally, a positive correlation was observed between donepezil and the MAPK signaling pathway, which is known to promote neuronal growth and proliferation.^[71]^ Donepezil was also observed to significantly elevate protein levels of PINK1 (which is associated with cytoprotection against mitochondrial dysfunction) in hippocampal tissue. This finding has been further confirmed by Western blotting.^[72]^ Another commonly used drug is galantamine, which acts as a nicotinic allosteric potentiating ligand. This enables it to modulate nicotinic cholinergic receptors, thereby increasing acetylcholine release. Additionally, galantamine acts as an acetylcholinesterase inhibitor.^[73]^ Memantine is an antagonist of the excitatory amino acid receptors, which improves cognitive, learning, and memory functions in patients with moderate-to-severe Alzheimer’s disease by antagonizing the excitatory toxicity of glutamate. Western blotting results have demonstrated that memantine nitrate-12 (one of the novel compounds derived from memantine) may exert neuroprotective effects through the inhibition of the extracellular signal-regulated kinase pathway and the activation of the phosphatidylinositol 3-kinase (PI3K)/protein kinase B (Akt) pathway.^[74]^ In a related study, memantine nitrate-06 pretreatment was demonstrated to suppress the PI3-K/Akt/GSK-3b pathway, thereby inhibiting glutamate-induced excitotoxicity and blocking glutamate-induced Ca2+ endocytosis.^[75]^ The current treatments for Alzheimer’s disease modify symptoms but do not cure the disease. For example, the acetylcholinesterase inhibitors and NMDA receptor antagonists mentioned above only improve the symptoms of cognitive and behavioral changes. Based on this, recent decades have seen attempts to develop new drugs for disease-modifying therapies aimed at stopping the clinical progression of Alzheimer’s disease at its roots.^[76]^ Immunotherapy is regarded as one of the most promising avenues in disease-modifying therapies for Alzheimer’s disease. Among the numerous disease-modifying drug targets, amyloid β and tau are considered the most promising. The emergence of aducanumab (a humanized recombinant monoclonal antibody directed against amyloid β) represents the first disease-modifying therapy to receive accelerated approval from the U.S. Food and Drug Administration for use in Alzheimer’s disease. The advent of aducanumab was met with considerable enthusiasm, but this initial optimism was not sustained. Subsequent reports indicated that patients experienced a range of adverse effects, predominantly amyloid-related imaging abnormalities (ARIA), including edema (ARIA-E) or microhemorrhages (ARIA-H). These findings were deemed less than satisfactory.^[77]^ The development of tau protein-targeted therapies for Alzheimer’s disease is still predominantly in phase II clinical trials,^[78]^ yet there is still considerable optimism regarding their potential to alter the course of the disease. This illustrates that disease-modifying therapies still have a significant journey ahead. In conclusion, it was found that symptomatic treatment programs such as donepezil only relieve symptoms, and often induce different degrees of side effects due to complex factors such as patient frailty and advanced age. Furthermore, various allopathic disease-modifying therapies are still in the exploratory stage. Therefore, an attempt is being made to explore new possibilities for the treatment of Alzheimer’s disease by starting from traditional Chinese medicines that have fewer side effects and multi-component and multi-target therapies. The PPI network diagram depicts the interactions among different proteins involved in cell cycle, energy metabolism, and signaling. The diagram illustrates the interactions among various proteins. AKT1, a serine/threonine protein kinase, is activated by insulin and various growth and survival factors. It serves as a crucial target of the PI3K-Akt signaling pathway, which regulates cell division, proliferation, apoptosis, and glucose metabolism.^[79]^ Abnormal brain insulin metabolism has long been considered a pathogenic mechanism of AD and has been experimentally demonstrated.^[80]^ AKT1 activation is not only related to learning and memory,^[81–83]^ but also normalizes insulin signaling. This enables the PI3K/Akt signaling pathway to operate normally, avoiding neuroinflammation, oxidative stress, and other pathological processes.^[84]^

STAT3 encodes a protein that belongs to the STAT family of proteins. It plays a crucial role in various cellular processes such as cell growth and apoptosis. STAT3 is activated by several cytokines, including IL-6 and IL-10, as well as growth factors such as EGF and FGF. Activation of STAT3 was found to be effective in rescuing hTau-induced synaptic dysfunction and memory impairment in mice in animal experiments.^[85]^ However, specific knockdown of STAT3 in AD model mice significantly reduced their brain amyloid levels and plaque load.^[86]^ This suggests that the role of STAT3 in Alzheimer’s disease is 2-fold. JUN is a transcription factor that regulates gene transcription in cells and influences BPs such as cell proliferation, differentiation, and apoptosis. Down-regulating JUN reduces the expression of inflammatory factors.^[87]^ Additionally, inhibiting c-Jun rescues neuronal death and damage in AD progenitor cells.^[88]^ The TP53-encoded p53 protein induces cell cycle arrest, apoptosis, senescence, DNA repair, or metabolic alterations. Aberrant alterations in p53 activity and AD are closely related. The first time p53 activity was found to be altered in AD was in skin fibroblasts from sporadic Alzheimer’s disease patients.^[89]^ Since then, numerous studies have shown that p53 dysregulation induces or exacerbates AD.^[90,91]^ The mTOR signaling pathway has been implicated in p53 activity in several studies. It is worth noting that this pathway is activated in early AD, as demonstrated by multiple studies.^[92,93]^ Therefore, p53 dysregulation may activate the mTOR signaling pathway and induce AD. Additionally, casp3, a cysteine-aspartic acid protease, maybe a potent target in early AD.^[94]^

The KEGG pathway enrichment analysis revealed that Polygonatum regulates the PI3K-Akt signaling pathway, pathways in cancer, pathways of neurodegeneration – multiple diseases, and lipid and atherosclerosis pathways to treat Alzheimer’s disease. For instance, the PI3K/AKT signaling pathway participates in various in vivo BPs, including apoptosis, inflammatory response, proliferation, and growth. Its activation inhibits GSK-3β and mTOR signaling, which, in turn, reduces tau protein phosphorylation.^[95]^ Previous experiments have shown that activation of metabotropic γ-aminobutyric acid receptors inhibits neuronal apoptosis and increases levels of SOD, GSH-Px, and CAT through the PI3K/Akt signaling pathway.^[96]^ This signaling pathway also regulates the amelioration of dysfunctional synaptic plasticity.^[97]^ In addition, a correlation has been found between estrogen loss and an increased risk of AD.^[98]^ Estrogen also protects nerves from toxic damage and reduces inflammatory signaling in neurons by regulating calcium flow.^[99]^ Numerous studies have confirmed the association between elevated cholesterol levels and an increased likelihood of developing AD. Specifically, elevated serum LDL levels are involved in the development of AD amyloid pathology.^[100,101]^ It is possible that vascular diseases, such as atherosclerosis, caused by abnormal cholesterol levels, are related to the pathology of AD. Research has shown that vascular dysfunction caused by atherosclerosis can disrupt the blood–brain barrier, induce inflammation, and impede β-amyloid clearance.^[102]^ The NF-κB signaling pathway and VEGF signaling pathway are also involved in the AD process, in addition to the pathways mentioned above.^[103–105]^ In conclusion, our network pharmacology analysis suggests that Polygonatum has the potential to treat Alzheimer’s disease through a multi-component, multi-target, and multi-pathway approach. For instance, baicalein targets TP53, CASP3, AKT1, and methylprotodioscin_qt simultaneously, while sibiricoside A_qt targets STAT3. Additionally, we found that 43 genes were enriched in pathways in cancer and 23 genes were enriched in the PI3 K/Akt signaling pathway.

Molecular docking is a technique used to predict ligand–receptor binding and calculate binding energies. We validated the docking of 10 active components of Polygonatum and 5 critical targets using molecular docking. The results demonstrated favorable binding energy for all 50 dockings, confirming our hypothesis regarding the potential of Polygonatum for treating Alzheimer’s disease. While network pharmacology aided in identifying active ingredients and corresponding targets of Polygonatum, and molecular docking validation yielded positive results, it is important to note that these findings were based on the analysis of numerous databases and network computer technology. Therefore, caution should be exercised when interpreting these results. Clinical studies are necessary to further validate the results, as we cannot guarantee the scientific validity of the database data or the accuracy of the computerized analyses.

## 5. Conclusions

In conclusion, this study used network pharmacology and computer simulation of molecular docking to demonstrate that the active ingredients of Polygonatum, such as beta-sitosterol, baicalein, and liquiritigenin, exert their therapeutic efficacy in treating Alzheimer’s disease by acting on targets such as AKT1, TP53, CASP3, JUN, STAT3, and others. Next, we will conduct experimental validation based on existing results to provide a practical solution for treating Alzheimer’s disease.

## Acknowledgments

We appreciate the support provided by the Jiangxi Provincial Administration of Traditional Chinese Medicine and Jiangxi Chuanqi Pharmaceutical Co.

## Author contributions

**Conceptualization:** Fang Chen, Zhihong Zhang.

**Data curation:** Liangliang Luo, Yao Pan, Fang Chen.

**Formal analysis:** Liangliang Luo, Fang Chen.

**Funding acquisition:** Zhihong Zhang.

**Investigation:** Liangliang Luo, Yao Pan.

**Methodology:** Fang Chen, Zhihong Zhang.

**Project administration:** Yao Pan, Zhihong Zhang.

**Supervision:** Liangliang Luo, Yao Pan, Fang Chen.

**Validation:** Liangliang Luo, Yao Pan.

**Visualization:** Liangliang Luo, Yao Pan.

**Writing – original draft:** Liangliang Luo.

**Writing – review & editing:** Yao Pan, Fang Chen, Zhihong Zhang.
